# Sex-associated differences in initial resection location, margin status, and re-excision in extremity leiomyosarcoma: a retrospective tertiary-center cohort study

**DOI:** 10.3389/fonc.2026.1980400

**Published:** 2026-10-19

**Authors:** Pia Weskamp, Sonja Verena Schmidt, Marius Drysch, Felix Reinkemeier, Mehran Dadras, Marcus Lehnhardt, Flemming Puscz, Christoph Wallner

**Affiliations:** 1 Department of Plastic Surgery and Hand Surgery, Burn Center, Sarcoma Center, BG University Hospital Bergmannsheil, Ruhr University Bochum, Bochum, Germany; 2 Clinic for Plastic and Reconstructive Surgery, Agaplesion Diakonieklinikum Hamburg, Hamburg, Germany

**Keywords:** leiomyosarcoma, re-excision, referral, selection bias, sex differences, soft tissue sarcoma, surgical margins

## Abstract

**Background:**

Sex-associated differences in early surgical care may occur within individual sarcoma entities. We examined recorded tumor characteristics, initial resection location, margin status, and re-excision in extremity leiomyosarcoma.

**Methods:**

This exploratory retrospective study included 79 patients with initial resection during 2000–2015 who ultimately received treatment at a tertiary sarcoma center. The primary endpoint was initial R1 versus R0 resection. Analyses used Firth penalized logistic regression and Bayesian marginal standardization; treatment-location, re-excision, and survival analyses were exploratory.

**Results:**

Initial R1 occurred in 34/46 men (73.9%) and 13/29 women (44.8%; absolute difference 29.1 percentage points; P = 0.015). Men more frequently underwent initial outside-center surgery (72.3% vs. 41.4%) and re-excision (82.6% vs. 50.0%). Recorded tumors <5 cm were more frequent in men (64.6% vs. 35.5%). The male-sex odds ratio decreased from 3.41 (95% CI 1.31–9.24) after age and site adjustment to 2.43 (0.82–7.41) after additional size and depth adjustment. The fully standardized difference was 16.6 percentage points (95% credible interval −2.5 to 37.4), compatible with no difference as well as a clinically substantial difference. Among patients with known location and R0/R1 status, R1 occurred after 37/44 outside-center and 9/29 index-center resections. All 47 patients with initial R1 underwent re-excision; definitive R0 was achieved in all 79 patients.

**Conclusion:**

Men in this selected, exploratory cohort had higher observed frequencies of outside-center resection, initial R1, and re-excision. Adjustment attenuated the association, with substantial residual uncertainty. These findings do not establish inequity, differential access, biological causation, or mediation and require prospective multicenter validation.

## Introduction

Complete resection is the basis of curative treatment for extremity soft-tissue sarcoma (1). A microscopically incomplete (R1) first resection may prompt tumor-bed re-excision and additional treatment burden (2–5). We selected initial R1 versus R0 as the primary endpoint because it captures the microscopic completeness of the first therapeutic operation, before subsequent surgery can obscure differences in the initial treatment course. Definitive margin status alone cannot capture this burden when all patients ultimately achieve R0. Initial R1 is nevertheless not a stand-alone measure of inappropriate care: anatomical constraints and deliberately planned close or positive margins may also influence margin status (1).

Sex-associated differences are increasingly recognized across cancer incidence, biology, treatment response, and prognosis (6). Reporting frameworks recommend direct statistical comparison by sex and clear separation of biological sex from gender-related social variables (7, 8). Whether sex is also associated with how patients enter and move through surgical cancer care has received much less attention.

Evidence for sex-associated differences in soft-tissue sarcoma care is limited. In a multicenter cohort of 728 patients, unplanned excision was more common among younger patients and those with smaller, superficial tumors, with a borderline association with male sex (P = 0.05); re-resection was also associated with greater reconstructive and radiotherapy burden (3). A nationwide Japanese registry study likewise associated unplanned excision with male sex and smaller, superficial tumors (9). Our study addresses a different, entity-specific question: whether recorded sex is associated with initial microscopic margin status and subsequent re-excision within extremity leiomyosarcoma. It does not estimate the incidence or effect of unplanned excision because planned versus unplanned outside-center surgery was not recorded. Prognostic tools such as Sarculator and Personalised Sarcoma Care (PERSARC) primarily estimate prognosis rather than the route to initial surgery or the need for another oncological operation (10, 11).

We examined the association between recorded sex and initial R1 resection in extremity leiomyosarcoma, alongside descriptive analyses of initial treatment location and re-excision. Successive adjustment assessed how the estimated association changed after inclusion of measured tumor characteristics. Exploratory analyses examined local recurrence and overall survival. The target is a within-cohort association among patients ultimately treated at the index center, not a causal effect of sex or a population estimate of inequity.

## Materials and methods

### Study design and cohort

This retrospective cohort included patients with histologically confirmed extremity leiomyosarcoma who underwent initial therapeutic resection between January 2000 and December 2015 and ultimately received treatment at the index tertiary sarcoma center. The institutional registry contained 82 extremity leiomyosarcoma records; three with initial resection before 2000 were excluded, leaving 79 patients. The eligibility interval refers to initial resection, including surgery outside the center. Diagnoses were confirmed by specialist sarcoma pathologists and reviewed by a multidisciplinary tumor board; histological terminology follows the World Health Organization classification (12). Truncal, retroperitoneal, and head-and-neck primaries were excluded. Reporting follows the Strengthening the Reporting of Observational Studies in Epidemiology (STROBE) statement (13). Patients successfully treated elsewhere without subsequent referral were not represented.

An exploratory comparison of initial outside-center resection across seven histologic groups with at least 20 patients was also performed during project development. Leiomyosarcoma was the only group with a nominal sex association, but this did not survive Benjamini–Hochberg adjustment (q = 0.125). That screen used a broader registry extract containing 82 leiomyosarcoma records; it is not independent validation of this 79-patient analysis. We therefore regard the present findings as hypothesis-generating and do not claim prospective prespecification of the entity-specific hypothesis.

### Endpoints and definitions

The primary endpoint was margin status at the initial therapeutic resection, dichotomized as R1 versus R0. Three patients with R2 resection (one woman and two men) were excluded from this comparison and included in a sensitivity analysis of initial non-R0 (R1/R2) versus R0. Initial margin status was missing in one woman. Thus, the primary denominator of 75 reflects three endpoint exclusions and one missing value, not four missing values. Secondary endpoints were initial resection location (index center versus elsewhere), tumor-bed re-excision, definitive margin, local recurrence, distant metastasis, and overall survival. Additional variables included age, maximum tumor diameter, depth, anatomic site, Fédération Nationale des Centres de Lutte Contre le Cancer (FNCLCC) grade, complex reconstruction, adjuvant radiotherapy, and postoperative complications.

The registry records where the initial resection took place but not whether it was planned or the type of outside institution. We therefore use the neutral term “initial resection outside the index center” and do not equate it with unplanned, inadequate, or non-specialist excision.

Maximum tumor diameter was recorded in four ordered categories (<5, 5–10, 10–15, and >15 cm) and was complete for all patients. The analytic registry did not systematically identify whether each value originated from preoperative imaging, the initial pathology specimen, or another source, or document the timing of measurement relative to referral. A uniform measurement source across initial treatment locations therefore cannot be established. We refer to recorded tumor size rather than size at first presentation to the center. Depth was classified relative to the investing fascia; anatomic site as upper versus lower extremity; and grade by FNCLCC. Reconstruction and complication status were taken from binary registry variables.

### Sex variable and terminology

Recorded sex was analyzed as female versus male, coded as 0 and 1, respectively. The source data did not include gender identity, gender roles, gender relations, hormone status, or other gender-related social variables. Accordingly, we use female/male or women/men to describe the recorded groups and the term “sex-associated” for statistical differences between them; we do not use “gender” as a synonym for “sex.” Reporting follows Sex and Gender Equity in Research (SAGER) recommendations (7).

### Conceptual framework and estimand

A conceptual directed acyclic graph (DAG) was developed during revision to clarify alternative biological, social, diagnostic, and selection mechanisms (**Figure 1**). It was not prespecified before the original analyses and is not presented as a validated causal model. Recorded sex is a grouping variable that may mark incompletely measured biological and social processes. True tumor extent may influence clinical suspicion and work-up, and may reflect both biological growth and diagnostic delay. The graph separates true extent from recorded size and depth because measurement source and specimen integrity may affect the recorded values. Initial treatment location, margin, and re-excision are temporally related endpoints, not interchangeable measures of care quality. Selection into the tertiary-center cohort may depend on these variables and on unmeasured patient or health-system factors. No measured adjustment set is claimed to identify a total or direct causal sex effect.

**Figure 1:**
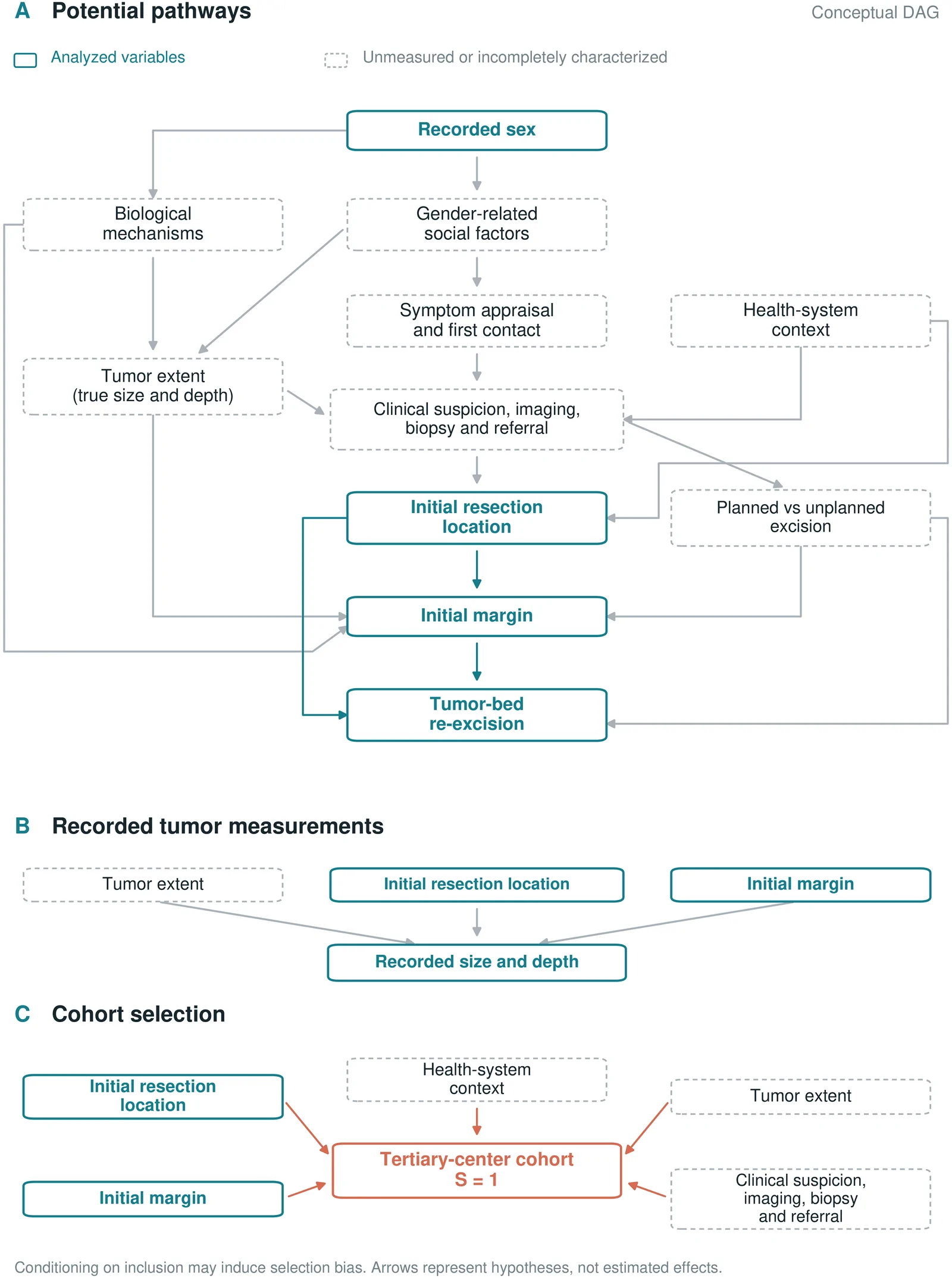
Partial conceptual directed acyclic graph developed *post hoc* during revision. **(A)** Hypothesized biological, social, diagnostic, and surgical pathways. Solid teal outlines denote analyzed variables; dashed gray outlines denote unmeasured or incompletely characterized variables. Arrows from recorded sex denote hypothesized sex-linked processes, not an intervention on sex. Symptom appraisal and first contact are separated from provider work-up and referral; whether excision was planned was not recorded. **(B)** True tumor extent is distinguished from recorded size and depth, which may also depend on measurement source and specimen integrity; the latter pathways concern size in particular. **(C)** The same variables may influence inclusion in the tertiary-center cohort (S = 1, coral outline). Every analysis conditions on inclusion, potentially inducing selection bias. Health-system context includes geography, provider specialty, and calendar period; calendar era was examined separately in sensitivity models. Age and anatomic site are omitted for readability. These diagrams state selected assumptions, do not identify a sufficient adjustment set, and do not rule out additional pathways. Validated referral timing was unavailable; selection at recurrence is an additional unresolved possibility.

### Statistical analysis

The primary comparison was initial R1 versus R0 by recorded sex. Categorical variables are reported as n/N (%), with absolute differences and risk ratios where appropriate, and compared using Fisher exact tests. Continuous variables are summarized as median (interquartile range, using linear interpolation) and compared using the Mann–Whitney U test. Each analysis used its evaluable denominator; missing values were not imputed. Missingness was tabulated by sex and initial treatment location. The registry did not document the reasons for the incomplete endpoint entries, and missingness was not assumed to be random. The focused analyses, including the principal endpoint, are exploratory; their P values are unadjusted for multiplicity. The separate histology screen is described above with its multiplicity-adjusted q value.

Adjusted analysis of the primary endpoint used Firth penalized logistic regression (14, 15). The four original models comprised sex alone; sex, age, and anatomic site; additional tumor size; and additional depth. Age was continuous and tumor-size category was entered as an ordered score from 1 to 4; all four models included the same 75 patients. We report odds ratios, profile penalized-likelihood 95% confidence intervals, and penalized likelihood-ratio P values. These are conditional associations within the selected cohort. Age and site provide covariate standardization; size and depth are measured tumor characteristics that may themselves lie downstream of sex-related biological or healthcare processes. Their inclusion does not define a causal direct effect. Changes in conditional odds ratios across models are descriptive and are not decomposed into confounding, mediation, or other components. Neither these changes nor changes in standardized risk differences identify a proportion mediated or explained.

To describe the observed sequence directly, we cross-tabulated initial treatment location, initial margin status, and re-excision by sex, retaining R2 and missing categories. No formal mediation analysis was performed. To explore calendar time, initial resection year was summarized by sex and a binary era term (2000-2007 versus 2008-2015) was added separately to the age/site-adjusted and fully adjusted Firth models. The equally long periods provide a coarse summary of calendar time; this split was not prospectively prespecified. Crude differences were also reported within each era.

Bayesian marginal standardization complements the Firth odds ratios by expressing the association as an absolute probability difference. It does not provide independent validation or overcome missing covariates or selection bias. The Bayesian analysis is used for estimation on this interpretable scale, with the full uncertainty interval reported alongside the point estimate.

For marginal standardization, logistic models contained sex, age, and site, with additional size and depth in the expanded model; both used the same 75 patients. All predictors, including binary indicators and the ordered size score from 1 to 4, were centered and divided by their cohort SD (divisor n). Independent normal priors had mean zero and SD 2.5 for these coefficients and SD 10 for the intercept. The analysis was rerun during revision using a fully documented implementation: four random-walk Metropolis chains, each with 10,000 warm-up and 40,000 retained iterations, without thinning. Gaussian proposals used the inverse Hessian at the posterior mode and were fixed during sampling; implementation details and seeds are given in **Supplementary Methods S5** and the accompanying code. At each posterior draw, predictions under the two sex indicators were averaged over the observed covariate distribution. We report the posterior median and equal-tailed 95% credible interval of the male–female probability difference. This is a model-based association, not an intervention effect of changing sex. Split R-hat and autocorrelation-based effective sample size were evaluated for all coefficients and the standardized difference.

Local recurrence was analyzed using cumulative incidence with death without prior recurrence as a competing event (16) and cause-specific Cox regression. Overall survival used Kaplan–Meier estimation and Cox regression. The registry-recorded survival intervals were anchored to initial resection. Follow-up was estimated by reverse Kaplan–Meier using the overall-survival interval, treating death as censoring and last contact in living patients as an event (17). The simple median observed interval is reported separately in **Table 1**. With 18 local recurrences and 21 deaths, prognostic models were restricted to at most two covariates. No equivalence testing was performed. Revision analyses used Python 3.12.14, NumPy 2.3.5, pandas 2.2.3, SciPy 1.17.0, pyreadstat 1.3.6, and lifelines 0.30.3. Firth estimation, posterior sampling, and the cumulative-incidence estimator were implemented in the accompanying Python code; Kaplan–Meier and Cox estimation used lifelines. Validated referral dates were unavailable for delayed-entry analysis.

**Table 1:** Patient, tumor, treatment, and outcome characteristics by sex.

Characteristic	Women (n = 31)	Men (n = 48)	Difference (pp)	P value
A. Patient and recorded tumor characteristics				
Age at resection, years	61 (54–68)	62 (50–67)	–	0.900
Maximum diameter <5 cm	11/31 (35.5)	31/48 (64.6)	+29.1	0.020
Deep (subfascial) tumor	14/31 (45.2)	12/48 (25.0)	−20.2	0.087
Upper extremity	8/31 (25.8)	10/48 (20.8)	−5.0	0.784
FNCLCC grade 3	13/31 (41.9)	15/48 (31.2)	−10.7	0.347
ASA class III or IV	4/31 (12.9)	7/48 (14.6)	+1.7	1.000
B. Initial surgical treatment				
Initial R1 resection	13/29 (44.8)	34/46 (73.9)	+29.1	0.015
Initial non-R0 (R1 or R2)	14/30 (46.7)	36/48 (75.0)	+28.3	0.016
Initial resection outside index center	12/29 (41.4)	34/47 (72.3)	+31.0	0.009
C. Sarcoma-center treatment				
Re-excision of the tumor bed	14/28 (50.0)	38/46 (82.6)	+32.6	0.004
Definitive R0 resection	31/31 (100.0)	48/48 (100.0)	0.0	not estimable
Complex reconstruction	6/31 (19.4)	16/48 (33.3)	+14.0	0.207
Adjuvant radiotherapy	12/31 (38.7)	14/48 (29.2)	−9.5	0.464
Any complication	14/31 (45.2)	17/48 (35.4)	−9.7	0.480
D. Oncological outcome				
Local recurrence	11/31 (35.5)	7/48 (14.6)	−20.9	0.053
Distant metastasis	5/31 (16.1)	10/48 (20.8)	+4.7	0.771
Death, any cause	8/31 (25.8)	13/48 (27.1)	+1.3	1.000
Observed follow-up, months	51 (18–91)	53 (19–90.5)	–	0.968

Data are median (interquartile range) or n/N (%). Differences are male minus female in percentage points. The R1/R0 comparison excludes three R2 cases (one woman, two men) and one woman with missing initial margin. Initial location is missing in two women and one man; re-excision in three women and two men. All other displayed denominators are specified in the table. P values are Fisher exact or Mann–Whitney U tests; all focused comparisons are exploratory and unadjusted for multiplicity. Quantiles use linear interpolation. Observed follow-up is the simple median recorded survival/contact interval, not the reverse Kaplan–Meier estimate. ASA, American Society of Anesthesiologists; FNCLCC, Fédération Nationale des Centres de Lutte Contre le Cancer; R0, microscopically negative; R1, microscopically positive; R2, macroscopically positive margin.

### Use of generative artificial intelligence

ChatGPT (OpenAI, https://chatgpt.com) and Claude (Anthropic, https://claude.ai) assisted language editing, manuscript restructuring, statistical-code development and review, and figure preparation. All four figures were redrawn using AI-assisted code. Exact model versions were not consistently recorded. The authors retain responsibility for the final text, references, analyses, and figures.

### Ethics statement

The study was approved by the ethics committee of the Ruhr University Bochum (approval number 20-7126) and conducted in accordance with the Declaration of Helsinki. Written informed consent for data collection and analysis was obtained from all patients.

## Results

### Cohort

The cohort comprised 31 women and 48 men. Median age was 62 years (interquartile range 52–68). Tumors arose in the lower extremity in 61/79 patients (77.2%), were superficial in 53/79 (67.1%), and were FNCLCC grade 3 in 28/79 (35.4%). Median follow-up estimated by reverse Kaplan–Meier was 72 months overall, 72 months in women, and 74 months in men. The median observed survival/contact interval was 51 months (interquartile range 18–91.5). Eighteen local recurrences and 21 deaths occurred (**Table 1**).

### Initial surgical treatment differed by sex

Initial R1 resection occurred in 34/46 men (73.9%) and 13/29 women (44.8%), an absolute difference of 29.1 percentage points (risk ratio 1.65, 95% CI 1.06–2.56; P = 0.015) (**Figure 2A**). Including the three R2 resections gave the same result: initial non-R0 in 36/48 men (75.0%) and 14/30 women (46.7%; P = 0.016).

**Figure 2:**
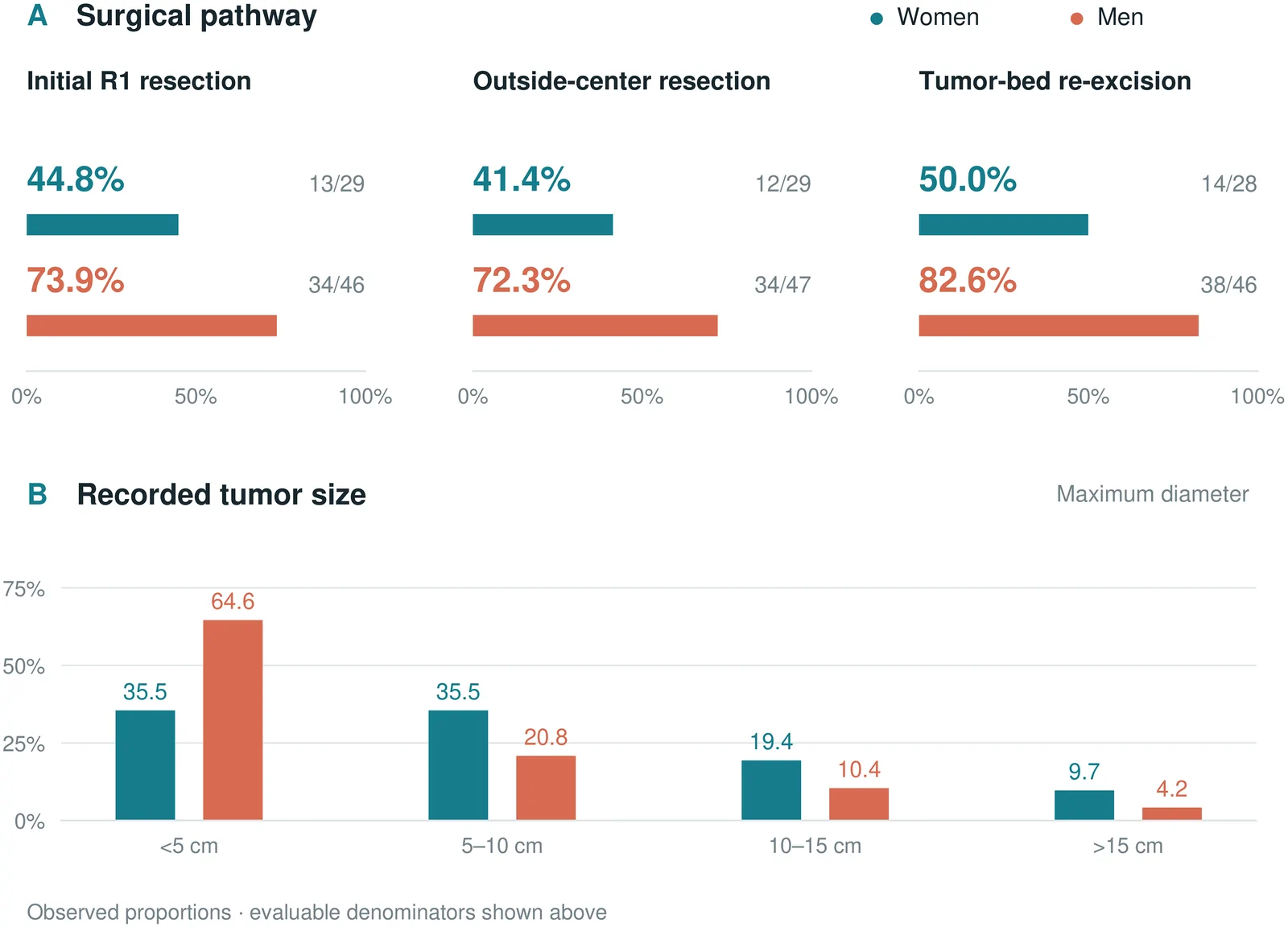
Observed sex-associated differences. **(A)** Initial R1, outside-center resection, and tumor-bed re-excision, with exact evaluable denominators. Teal bars denote women and coral bars denote men. Percentages and evaluable counts are labeled directly. **(B)** Recorded tumor-size distribution among 31 women and 48 men. These are unadjusted descriptive proportions; outside-center resection is not synonymous with unplanned excision. The primary R1 denominator excludes R2 and missing initial margin.

Initial outside-center resection occurred in 34/47 men (72.3%) and 12/29 women (41.4%; difference 31.0 percentage points; exploratory P = 0.009). Re-excision occurred in 38/46 men (82.6%) and 14/28 women (50.0%; difference 32.6 points; exploratory P = 0.004). Definitive R0 was achieved in all 79 patients. These are related observations within the same cohort, not independent replications (**Figure 2A**).

### Observed location, margin, and re-excision sequence

Among patients with known initial location and R0/R1 status, R1 occurred in 37/44 outside-center resections (84.1%) and 9/29 index-center resections (31.0%). The corresponding proportions were 9/12 versus 4/16 in women and 28/32 versus 5/13 in men. All 47 patients with initial R1 underwent re-excision: 37 after outside-center surgery, nine after index-center surgery, and one with missing initial location. The remaining five recorded re-excisions followed three R2 and two R0 resections. **Table 2** shows every location–margin–re-excision combination by sex, including missing values; it describes co-occurrence and recorded temporal order without estimating mediation.

**Table 2:** Initial location, margin status, and subsequent re-excision by sex.

Sex	Initial location	Initial margin	n	Re-excision: yes	Re-excision: no	Re-excision: missing
Women	Index center	R0	12	0	11	1
Women	Index center	R1	4	4	0	0
Women	Index center	R2	1	1	0	0
Women	Outside center	R0	3	0	2	1
Women	Outside center	R1	9	9	0	0
Women	Missing location	R0	1	0	1	0
Women	Missing location	Missing margin	1	0	0	1
Men	Index center	R0	8	0	6	2
Men	Index center	R1	5	5	0	0
Men	Outside center	R0	4	2	2	0
Men	Outside center	R1	28	28	0	0
Men	Outside center	R2	2	2	0	0
Men	Missing location	R1	1	1	0	0

All 79 patients are represented; each row is a mutually exclusive combination. Re-excision denotes the recorded subsequent tumor-bed procedure. R2 and missing categories are retained. The table describes observed treatment sequences, not causal effects or mediation.

### Missing endpoint data

Initial margin was missing in 1/31 women (3.2%) and 0/48 men; initial location in 2/31 women (6.5%) and 1/48 men (2.1%); and re-excision in 3/31 women (9.7%) and 2/48 men (4.2%). Re-excision status was missing in 3/30 patients with index-center initial surgery, 1/46 with outside-center surgery, and 1/3 with unknown initial location. The single missing initial margin was in the unknown-location group. Reasons for these incomplete entries were not documented (**Supplementary Table S3**).

### Recorded tumor characteristics and adjusted associations

Recorded tumors <5 cm were more frequent in men: 31/48 (64.6%) versus 11/31 in women (35.5%; exploratory P = 0.020). Deep tumors occurred in 12/48 men (25.0%) and 14/31 women (45.2%; exploratory P = 0.087). The full size distribution is shown in **Figure 2B**; age, anatomic site, grade, and other characteristics are reported in **Table 1**.

In Firth models, the male-sex odds ratio for initial R1 was 3.37 (95% CI 1.30–9.10; P = 0.012) without adjustment and 3.41 (1.31–9.24; P = 0.011) after age and site adjustment. It decreased to 2.54 (0.91–7.29; P = 0.076) after adding tumor size and to 2.43 (0.82–7.41; P = 0.110) after adding depth. The models containing measured tumor characteristics therefore produced attenuated, imprecise estimates with intervals including the null (**Figure 3A**).

**Figure 3:**
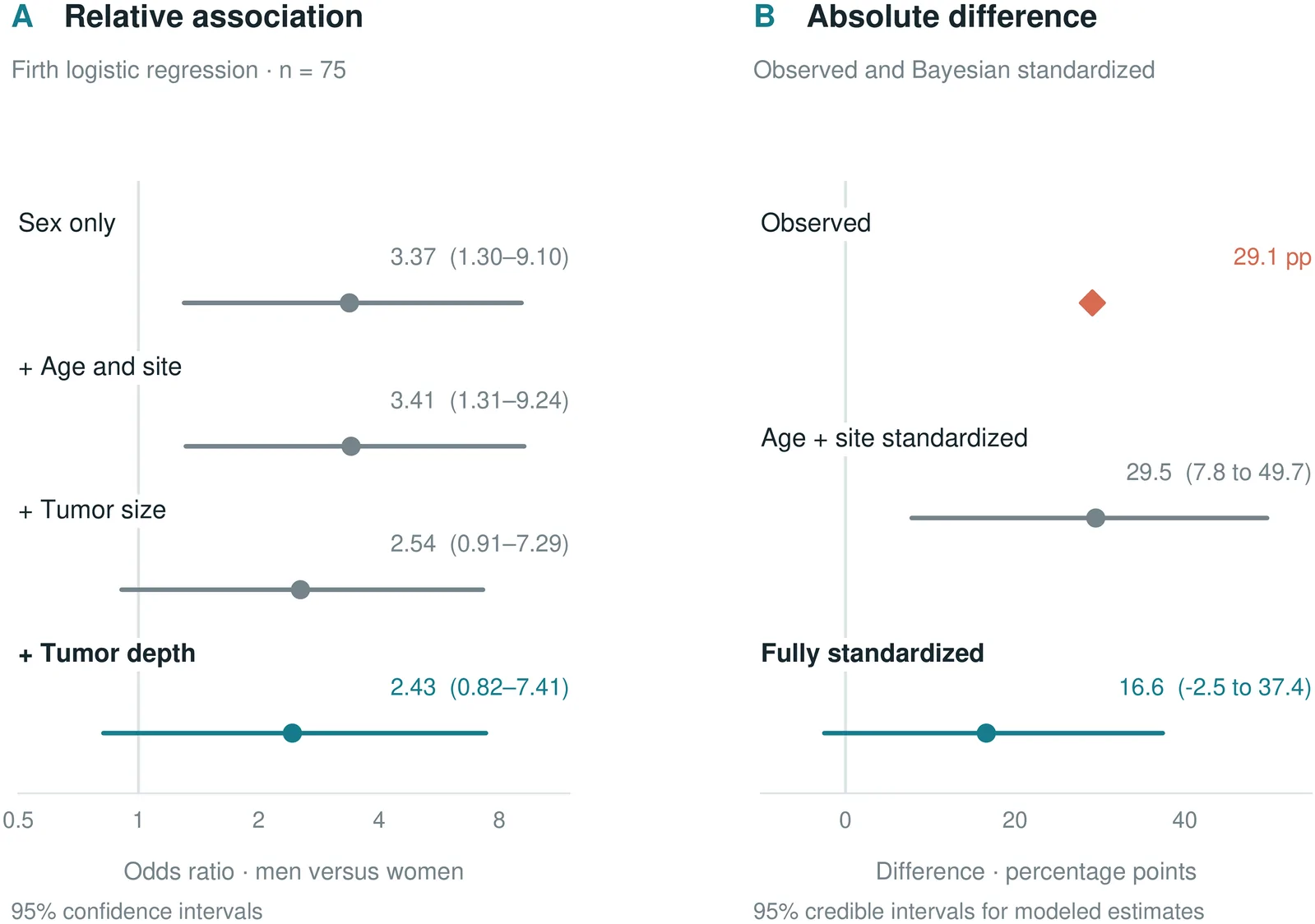
Conditional and standardized associations with initial R1. **(A)** Successive Firth odds ratios for male versus female sex with profile penalized-likelihood 95% confidence intervals; all models include the same 75 patients. **(B)** Observed male–female difference (coral diamond, without an interval) and Bayesian standardized differences (circles, posterior median and 95% credible interval). Observed and standardized estimates are explicitly distinguished. Teal highlights the fully adjusted or standardized model, including age, site, recorded tumor size, and depth; gray denotes less adjusted models. Neither attenuation nor standardization identifies mediation or a causal sex effect.

Initial resection year was 2006 (interquartile range 2003.5–2009.5) in women and 2007 (2004.75–2010) in men. Sixteen of 31 women (51.6%) and 26/48 men (54.2%) underwent initial resection in 2000–2007. Adding era yielded male-sex odds ratios of 3.36 (95% CI 1.30–9.04) for the age/site-adjusted model and 2.40 (0.81–7.30) for the fully adjusted model. Crude male–female R1 differences were 35.0 percentage points in 2000–2007 and 22.7 in 2008–2015; outside-center resection differences were 48.4 and 13.3 points, respectively (**Supplementary Table S4**). The small strata preclude firm conclusions about temporal heterogeneity. The limited change after era adjustment does not establish temporal stability (**Supplementary Tables S1, S2**).

On the absolute scale, age/site standardization yielded a male–female difference in R1 probability of 29.5 percentage points (95% credible interval 7.8–49.7). After inclusion of size and depth, the difference was 16.6 points (−2.5 to 37.4). The latter interval is compatible with a small excess in women, no difference, or a substantial excess in men. The estimates should therefore not be interpreted as establishing a residual sex effect. Across the two rerun models, maximum split R-hat was 1.001 and minimum effective sample size was 7,645; the acceptance fraction ranged from 0.300 to 0.317. These diagnostics support numerical convergence, not validity of the model assumptions. **Figure 3B** separates the observed difference from these standardized estimates; no percentage explained or mediated is reported.

### Exploratory oncological outcomes

Five-year cumulative incidence of local recurrence was 26.1% after initial R1 and 25.9% after initial R0 (10/47 and 7/28 events; cause-specific hazard ratio 0.79, 95% CI 0.30–2.08; P = 0.637; **Figure 4A**). Distant metastasis occurred in 11/47 patients after R1 (23.4%) and 4/28 after R0 (14.3%; P = 0.388). Five-year overall survival was 77.3% after R1 and 79.3% after R0 (hazard ratio 0.78, 95% CI 0.31–1.96; P = 0.599; **Figure 4B**). These wide intervals do not establish equivalence.

**Figure 4:**
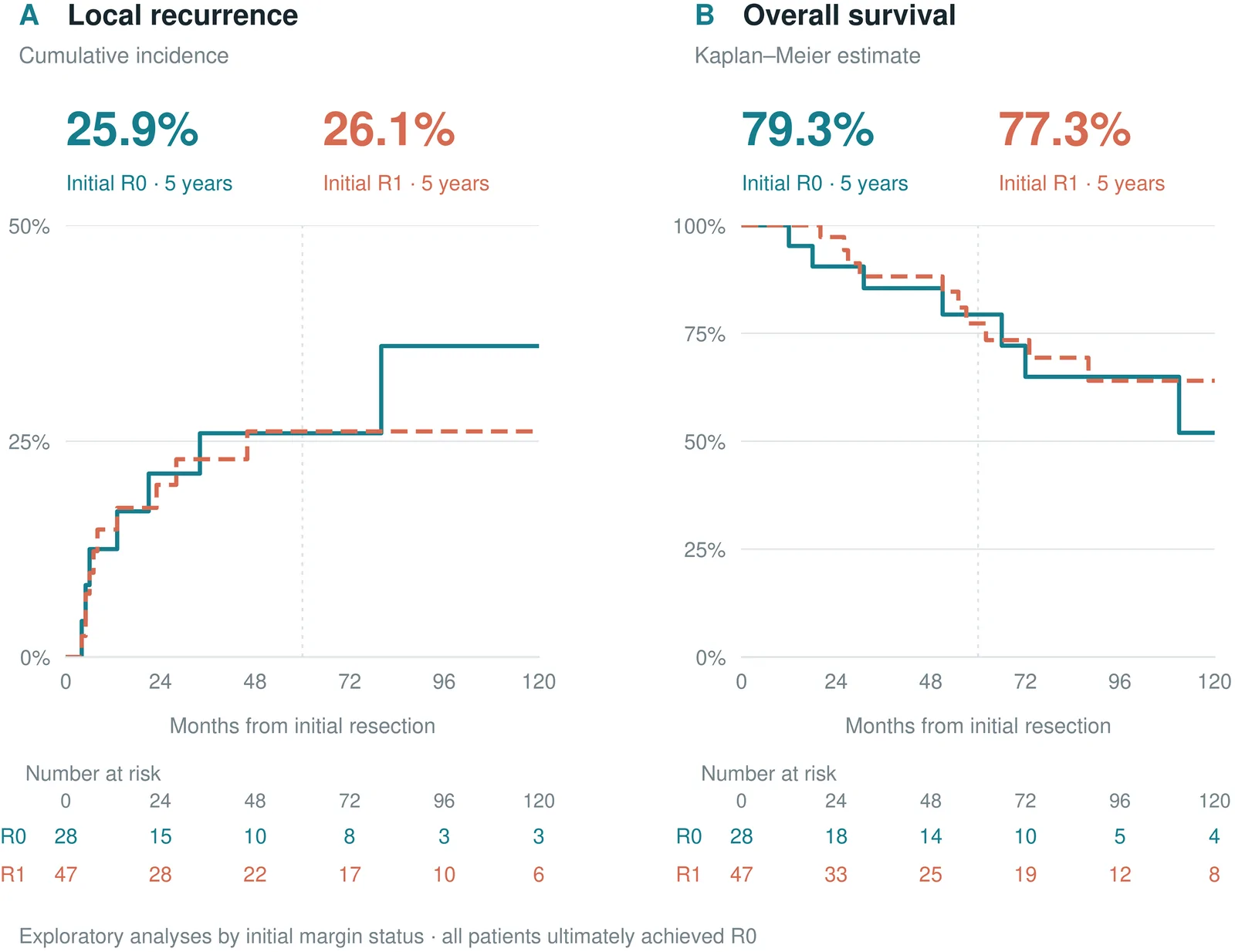
Exploratory oncological outcomes by initial margin. The analysis includes 75 patients; three R2 cases and one missing initial margin were excluded. **(A)** Cumulative incidence of local recurrence, treating death without prior recurrence as a competing event. **(B)** Kaplan–Meier overall survival. Teal solid curves denote initial R0 and coral dashed curves initial R1. The corresponding 5-year estimates are displayed above each panel; the vertical dotted line marks 60 months. Numbers at risk are shown below each panel. Time is measured from initial resection. Curves are descriptive and shown to 120 months; all available follow-up contributes to the analyses. Subsequent definitive R0 was achieved in every patient, and the comparisons do not establish equivalence. Delayed entry at referral was not modeled; survival to referral and possible referral at recurrence may distort these descriptive comparisons.

Local recurrence occurred in 11/31 women (35.5%) and 7/48 men (14.6%; P = 0.053). Distant metastasis and death comparisons yielded P = 0.771 and P = 1.000, respectively. In a model containing initial margin and sex, the local-recurrence hazard ratios were 1.18 for initial R1 (95% CI 0.42–3.34; P = 0.759) and 0.37 for male sex (0.13–1.04; P = 0.059). For overall survival, the corresponding estimates were 0.76 (0.28–2.08; P = 0.595) and 1.07 (0.40–2.86; P = 0.898). All comparisons are exploratory. Because every patient ultimately achieved definitive R0, these results describe a cohort receiving specialist treatment and re-excision where indicated; they cannot show that initial R1 is harmless.

## Discussion

In this selected tertiary-center cohort, men had higher observed frequencies of initial outside-center surgery, R1 resection, and re-excision, with absolute differences of approximately 30 percentage points. The individual-level cross-tabulation confirms that initial R1 and subsequent re-excision co-occurred: all 47 patients with R1 underwent re-excision, including 37 whose first resection was outside the index center. This supports the description of an observed treatment sequence, but does not establish that outside-center surgery caused margin positivity or that the between-sex difference represents inequity.

Adding recorded size and depth reduced the estimated odds ratio from 3.41 to 2.43 and the standardized difference from 29.5 to 16.6 percentage points. The fully adjusted Firth interval included an odds ratio of one, and the standardized absolute difference of 16.6 percentage points had a credible interval from −2.5 to 37.4. These findings support considerable uncertainty about both the presence and magnitude of a residual association. Neither the change in the odds ratio nor the change in the standardized difference identifies mediation. Accordingly, no proportion explained or mediated is inferred.

The main clinical observation is additional surgical burden. Re-excision of a previously operated tumor bed can require wider tissue removal and additional reconstructive or radiotherapeutic treatment (3, 5). However, we did not demonstrate that these downstream burdens were caused by sex or initial treatment location. Definitive R0 was achieved in every patient, and the exploratory survival comparisons were imprecise. The absence of a detectable survival association after the completed treatment course is not evidence of equivalence between initial R1 and R0.

Local recurrence was recorded more often in women than men (11/31 versus 7/48; exploratory P = 0.053). In the model containing initial margin and sex, the male-sex hazard ratio was 0.37 (95% CI 0.13–1.04). This direction contrasts with the initial-margin difference. The descriptive comparison includes 18 events, while the margin-adjusted model includes 17 events among 75 patients; differences in tumor characteristics, treatment, selection, or chance remain possible explanations.

Small or superficial sarcomas may resemble benign lesions and generate less clinical suspicion, potentially altering imaging, biopsy, referral, and the site of initial surgery. The phenotype seen more frequently in men here is consistent with the profile reported after unplanned excision in a heterogeneous multicenter cohort (3). A recent Japanese multicenter study also documented variation in the specialties undertaking unplanned excision and in preoperative MRI use (18). These observations support the plausibility of a recognition and referral mechanism; they do not show that it operated in our patients. The same recorded tumor phenotype could reflect biology, the timing or source of measurement, diagnostic decisions, or selection into this cohort.

The conceptual DAG makes these competing interpretations explicit (**Figure 1**). Recorded sex may mark biological and social processes; it does not measure gender identity, roles, health literacy, or healthcare behavior. Tumor size and depth need not be conventional confounders and could lie on pathways linking sex-related processes to management. Likewise, education and socioeconomic position could be components of a social pathway rather than adjustment variables to be included automatically. Symptom duration, first healthcare contact, preoperative diagnostic impression, referring specialty, imaging/biopsy sequence, planned versus unplanned excision, education, socioeconomic position, geographical accessibility, and detailed comorbidity were not jointly characterized. Their absence is a central limit on interpretation, not only a motivation for future work. No claim about sex differences in help-seeking can be supported.

Health-system context also matters. The French nationwide NETSARC+ analysis found associations between clinical characteristics and early specialist assessment, a smaller association with distance, and no association with municipality-level social deprivation (19). Access therefore cannot be inferred from sex alone. Our 2000–2015 cohort spans changes in imaging, referral awareness, multidisciplinary practice, and centralization. Adding a binary era term changed the pooled sex estimate little, but crude differences were larger in the earlier era, particularly for outside-center surgery (48.4 versus 13.3 percentage points). These small-stratum descriptions do not establish an interaction, and a binary term cannot capture changing referral rules or institution-specific practice. Contemporary multicenter data are needed before generalizing this historical pattern.

Referral selection is a fundamental threat to internal interpretation. Patients with successful outside R0 surgery who never reached the center are absent. If inclusion depends on initial margin, tumor characteristics, treatment location, and sex-linked patient or health-system factors, conditioning on tertiary-center treatment may induce associations through selection. This could change the magnitude or even direction of the observed sex association; adjustment for measured tumor characteristics does not correct it. Additional limitations are the small sample, wide intervals, incomplete endpoint records, lack of uniform documentation of size-measurement provenance, and absence of external validation. Missingness differs numerically by sex and pathway, and its causes cannot be established from the registry. The cohort overlaps a previous institutional somatic leiomyosarcoma series that examined prognosis but not the present sex-specific surgical endpoints (20).

Measurement provenance also affects interpretation of adjustment. If diameter was derived from an initial resection specimen, recorded size could depend on the location and technique of surgery or on specimen integrity, rather than solely on pretreatment extent. Differential measurement could bias the sex-associated estimate in either direction. Models including recorded size and depth are therefore descriptive and should not be treated as necessarily less biased estimates of a causal effect.

Patients initially operated on elsewhere had to survive until referral to enter this cohort. Analyses beginning at initial resection without delayed entry may therefore overestimate survival or distort comparisons involving initial location and margin. A validated date of first index-center contact and the relation of any recurrence to that contact could not be established from the analytic extract, so delayed entry was not modeled. Recurrence-free survival before referral cannot be assumed; referral triggered by recurrence would create additional outcome-dependent selection.

The contribution is therefore an exploratory, entity-specific description of recorded treatment differences and their uncertainty. The histology-wide screen did not meet its multiplicity-adjusted threshold, and the present cohort is not an independent validation; data-dependent focus and selective emphasis remain concerns. Prospective studies should capture patients from first clinical assessment, including those who remain outside specialist centers, and record diagnostic sequence, measurement provenance, planned versus unplanned surgery, and social and geographical context. Such a design would permit evaluation of modifiable determinants without interpreting recorded sex as a mechanism.

## Conclusion

Within this historical tertiary-center cohort, men more frequently underwent initial outside-center resection, R1 resection, and re-excision. Adjustment for measured tumor characteristics attenuated the association, and the fully adjusted intervals included the null. The observed treatment sequence is clinically relevant but does not establish inequity, differential specialist access, biological causation, or mediation. Referral selection may materially influence the findings. Prospective multicenter investigation beginning before initial surgery is warranted.

## Data Availability

The results supporting this study are presented in the article and **Supplementary Material**. The de-identified participant-level dataset and analysis code are available from the corresponding authors on reasonable request, subject to institutional and data-protection requirements.
