# Supplementary material for: Sex-associated differences in initial resection location, margin status, and re-excision in extremity leiomyosarcoma: a retrospective tertiary-center cohort study
Source: Front Oncol. 2026 Oct 19;16:1980400. doi: 10.3389/fonc.2026.1980400 (PMC13724375; doi:10.3389/fonc.2026.1980400)
Supplement: Supplementary file 1 [file DataSheet1.docx]

Supplementary material

Sex-associated differences in initial resection location, margin status, and re-excision in extremity leiomyosarcoma: a retrospective tertiary-center cohort study

Manuscript 1980400 | Revision of September 2026

This supplement reports calendar-period sensitivity analyses, endpoint missingness, era-specific observed differences, and the reproducible Bayesian rerun. All analyses use the same 79-patient cohort; R1 versus R0 models use the same 75 patients as the original submission.

# Table S1 Calendar period and follow-up

| **Characteristic** | **Women (n = 31)** | **Men (n = 48)** | **Overall (n = 79)** |
| --- | --- | --- | --- |
| Initial resection year, median (IQR) | 2006 (2003.5–2009.5) | 2007 (2004.75–2010) | — |
| 2000–2007, n/N (%) | 16/31 (51.6) | 26/48 (54.2) | 42/79 (53.2) |
| 2008–2015, n/N (%) | 15/31 (48.4) | 22/48 (45.8) | 37/79 (46.8) |
| Reverse Kaplan–Meier median follow-up, months | 72 | 74 | 72 |
| Simple median observed interval, months | 51 | 53 | 51 |

IQR, interquartile range. The Mann–Whitney comparison of initial resection year yielded exploratory P = 0.411. Reverse Kaplan–Meier uses the registry overall-survival interval, with death censored and last contact in living patients as the event. This differs from the simple median observed survival/contact interval. No claim of equivalent temporal distributions is made. Quantiles use linear interpolation; fractional calendar years are interpolated percentiles, not individual resection dates.

# Table S2 Successive Firth models and calendar-era sensitivity

| **Model** | **n** | **Male-sex OR** | **95% CI** | **P** |
| --- | --- | --- | --- | --- |
| Sex only | 75 | 3.37 | 1.30–9.10 | 0.012 |
| Sex + age + site | 75 | 3.41 | 1.31–9.24 | 0.011 |
| Sex + age + site + size | 75 | 2.54 | 0.91–7.29 | 0.076 |
| Sex + age + site + size + depth | 75 | 2.43 | 0.82–7.41 | 0.110 |
| Sex + age + site + era | 75 | 3.36 | 1.30–9.04 | 0.012 |
| Sex + age + site + size + depth + era | 75 | 2.40 | 0.81–7.30 | 0.112 |

Odds ratios compare men with women for initial R1 versus R0. Age is continuous; site is upper versus lower extremity; tumor-size category is an ordered score (1–4); depth is subfascial versus superficial. Era is 2008–2015 versus 2000–2007, based on the initial resection date. The equal-duration split was not prospectively prespecified. Confidence intervals use profile penalized likelihood and P values use penalized likelihood-ratio tests. Models with era are exploratory sensitivity analyses. They do not account for unmeasured diagnostic or referral processes, selection into the cohort, or more complex historical changes.

# Table S3 Missing endpoint data

| **Variable** | **Women missing** | **Men missing** | **Overall missing** |
| --- | --- | --- | --- |
| Initial margin | 1/31 (3.2) | 0/48 (0.0) | 1/79 (1.3) |
| Initial resection location | 2/31 (6.5) | 1/48 (2.1) | 3/79 (3.8) |
| Re-excision | 3/31 (9.7) | 2/48 (4.2) | 5/79 (6.3) |

## Missingness by initial treatment location

| **Initial location** | **Patients** | **Initial margin missing** | **Re-excision missing** |
| --- | --- | --- | --- |
| Index center | 30 | 0/30 | 3/30 (10.0%) |
| Outside center | 46 | 0/46 | 1/46 (2.2%) |
| Unknown | 3 | 1/3 (33.3%) | 1/3 (33.3%) |

The primary R1/R0 denominator of 75 is obtained by excluding three R2 cases and one missing margin from 79; R2 is not missing data. The non-R0 sensitivity comparison includes 78 patients. The reasons for incomplete registry entries were not documented, and no missing-at-random assumption is made. Table 2 in the main manuscript retains all missing combinations. The original initial-margin field was used consistently; an alternative similarly named field was not used to back-fill missing values without source adjudication.

# Table S4 Observed differences within calendar eras

| **Era** | **Endpoint** | **Women n/N (%)** | **Men n/N (%)** | **Difference (pp)** |
| --- | --- | --- | --- | --- |
| 2000–2007 | Initial R1 | 6/15 (40.0) | 18/24 (75.0) | 35.0 |
| 2000–2007 | Outside-center resection | 4/14 (28.6) | 20/26 (76.9) | 48.4 |
| 2008–2015 | Initial R1 | 7/14 (50.0) | 16/22 (72.7) | 22.7 |
| 2008–2015 | Outside-center resection | 8/15 (53.3) | 14/21 (66.7) | 13.3 |

Differences are men minus women. Denominators are evaluable records within each era. Initial R1 is compared with R0; R2 and missing margins are excluded. These small-stratum comparisons are exploratory and do not establish an interaction or temporal stability. Rounded differences were calculated from unrounded proportions.

## Exploratory histology screen

An exploratory comparison of initial outside-center resection across seven histologic groups with at least 20 patients was also performed during project development. Leiomyosarcoma was the only group with a nominal sex association, but this did not survive Benjamini–Hochberg adjustment (q = 0.125). That screen used a broader registry extract containing 82 leiomyosarcoma records; it is not independent validation of this 79-patient analysis. We therefore regard the present findings as hypothesis-generating and do not claim prospective prespecification of the entity-specific hypothesis.

# Methods S5 Reproducible Bayesian standardization

Both Bayesian models were rerun during revision using the implementation specified below. The updated estimates supersede the earlier results. Both models use the same 75 R0/R1 patients, with no missing covariates. Results describe the selected cohort.

For marginal standardization, logistic models contained sex, age, and site, with additional size and depth in the expanded model; both used the same 75 patients. All predictors, including binary indicators and the ordered size score from 1 to 4, were centered and divided by their cohort SD (divisor n). Independent normal priors had mean zero and SD 2.5 for these coefficients and SD 10 for the intercept. The analysis was rerun during revision using a fully documented implementation: four random-walk Metropolis chains, each with 10,000 warm-up and 40,000 retained iterations, without thinning. Gaussian proposals used the inverse Hessian at the posterior mode and were fixed during sampling; implementation details and seeds are given in Supplementary Methods S5 and the accompanying code. At each posterior draw, predictions under the two sex indicators were averaged over the observed covariate distribution. We report the posterior median and equal-tailed 95% credible interval of the male–female probability difference. This is a model-based association, not an intervention effect of changing sex. Split R-hat and autocorrelation-based effective sample size were evaluated for all coefficients and the standardized difference.

For each model, a BFGS optimization located the posterior mode. The proposal covariance was (2.38² / k) times the inverse negative-log-posterior Hessian at that mode, where k is the number of coefficients including the intercept. Proposals remained fixed; this rerun does not use adaptive sampling. Initial states were overdispersed around the mode. NumPy default_rng seeds were 1980400–1980403 for age/site and 1980500–1980503 for the expanded model. Code reports all centering means and SDs. Effective sample sizes use split chains and the initial-positive, monotone paired-autocorrelation sequence; R-hat is the ordinary split-chain statistic, not rank-normalized R-hat.

| **Model** | **Median difference (pp)** | **95% CrI** | **Max split R-hat** | **Min ESS** |
| --- | --- | --- | --- | --- |
| Age + site | 29.5 | 7.8 to 49.7 | 1.00045 | 11,081 |
| Age + site + size + depth | 16.6 | −2.5 to 37.4 | 1.00074 | 7,645 |

Each model has four chains with 40,000 retained draws per chain (160,000 draws per model). Acceptance fractions were 0.300–0.317 across chains. Diagnostics cover coefficients and standardized differences. Bayesian uncertainty does not account for classification error, measurement error, referral selection, model misspecification, or any data-dependent choice of study focus. The reported diagnostics assess numerical mixing only. Software: Python 3.12.14, NumPy 2.3.5, pandas 2.2.3, SciPy 1.17.0, pyreadstat 1.3.6, lifelines 0.30.3.
